# A simple mooring modification reduces impacts on seagrass meadows

**DOI:** 10.1038/s41598-019-55425-y

**Published:** 2019-12-27

**Authors:** Anna L. Luff, Emma V. Sheehan, Mark Parry, Nicholas D. Higgs

**Affiliations:** 1 University of Plymouth, School of Biological and Marine Sciences, Plymouth, PL4 8AA UK; 2 National Marine Aquarium, Community Seagrass Initiative, Plymouth, PL4 0LF UK; 3 Cape Eleuthera Institute, Rock Sound, EL-26029 Eleuthera Bahamas

**Keywords:** Conservation biology, Environmental impact

## Abstract

Moorings can have a detrimental impact on seagrass, fragmenting the meadows, resulting in the habitat degradation. To reduce contact of the moorings with the seabed we attached small floats along the chain of a traditional swing mooring and monitored the ecological impacts of this modified mooring, with reference to a standard swing mooring, in a seagrass meadow under high tidal influence. After three years, seagrass density surrounding the modified mooring was over twice as high as that of the standard mooring, with blade length surrounding the modified mooring also found to exceed that of the standard mooring. Seagrass-associated epifaunal species richness was twice as high surrounding the modified mooring compared to the standard mooring. Sediment composition was considerably finer at the modified mooring, indicative of increased disturbance surrounding the standard mooring. A simple modification to existing swing moorings can mitigate some of the impacts of moorings on seagrass meadows, whilst accommodating for tidal fluctuations. The scale of the differences observed between the mooring types demonstrates the susceptibility of seagrass meadows to damage from swing moorings. Given the ecological importance of these habitats, it is crucial that action is taken to reduce further degradation, such as that demonstrated here.

## Introduction

Shallow, sheltered coastal bays provide ideal conditions for the growth of temperate seagrass meadows, but are also attractive mooring and anchorage sites for boating communities. Anchoring and mooring causes physical disturbance to the seagrass that has a number of deleterious consequences. However, the ecological importance of seagrass habitat is widely recognized, and seagrasses are protected by law in many countries^1^. Therefore, it is often problematic for environmental managers to balance the needs of the maritime leisure industry and conservation obligations, especially when maritime safety is paramount. The most common solution is to provide fixed moorings that negate the need for anchoring, but moorings also cause lasting damage to seagrass^2^.

The most commonly used mooring system is the swing mooring, a system that consists of a sinker block on the seafloor, and a heavy chain reaching a surface buoy, on which the boat is secured. The buoy and chain pivot as the boat moves with the changing tide and wind, dragging the chain across the seafloor, resulting in scouring and the creation of ‘mooring scars’, circular areas of bare ground surrounding the mooring, which can be seen in satellite imagery. Impacts from mooring infrastructure on seagrass meadows have been widely studied, although few studies are undertaken in areas of increased tidal fluctuation, or focus on the seagrass species *Zostera marina*^2^.

Seagrass meadows provide key ecological services, these include sediment stabilization and natural coastal defenses during extreme weather, carbon sequestration, nutrient cycling, the provision of fish nurseries and enhancement to biodiversity^3^. Anthropogenic activities including anchoring, mooring, propeller scaring, vessel grounding and dredging have been found to negatively affect the rhizomes and bury seeds thus inhibiting germination and reduce the provision of these ecological services^3^. Impacts to seagrass can also result from extreme weather, invasive species, overgrazing and algal blooms^3^. Physical impacts to seagrass bed substrates can influence microbial communities within the sediments, often leading to the release of CO_2_ from blue carbon sinks in the meadow, acting as a contributor to global warming^4^. In addition to this, sediment disturbance can also result in the loss of seagrass meadow stability, leading to increased fragmentation of the meadow, erosion, and a reduction in sedimentation, often resulting in the decline of seagrass cover and a loss of resilience, leaving the seagrass meadows prone to impacts from other stressors^2,5^. Seagrass loss has also been found to effect associated fauna, with negative impacts observed on species density and richness, alongside changes to species assemblage^6,7^. Impacts on seagrass ecosystems are also expected to have an effect on local fisheries dependent on the high diversity of commercial species supported by seagrass meadows^5,8^. A study by Jackson *et al*.^9^ estimated that seagrass associated species contributed approximately 30–40% to the value of commercial fisheries landings, highlighting the economic value of seagrass meadows.

As an approach to reduce anthropogenic impacts on seagrass, various ‘environmentally friendly moorings’ or ‘eco-moorings’ have been designed to reduce the detrimental impacts of mooring chains on seagrass meadows. Eco-moorings are primarily designed to reduce chain abrasion on the seafloor, whilst ensuring a secure mooring for vessels in prevailing conditions. The moorings typically consist of two common features; a rode and buoy system designed to reduce contact and scouring of the seafloor, and an anchorage; both features vary in design across different moorings. The rode is often either rope, chain or an elastic tether, a preferred option in areas of increased tidal range. The anchorage can be a concrete block such as those used in swing moorings, or a substrate embedment anchor, which is often preferable due to its reduced ecological impact^10^.

A frequently used eco-mooring system is the Ezyrider design, this consists of a chain rode with an elastic riser system, and a displacement buoy that moves up and down a stainless-steel shaft with movement of the vessel. The system also uses ground weights as anchorage, although can be installed with an alternative ‘Offset Anchor System’ (a three-pronged structure) for more sensitive habitats such as seagrass meadows^10^.

An alternative eco-mooring system is the Seaflex mooring buoy, an elastic mooring system that can be used in conjunction with any anchorage, and if used alongside a seagrass friendly anchor could reduce scouring of the seafloor. An example of a seagrass friendly anchor is the Helix anchor, a corkscrew type substrate embedment anchor which boasts minimal disturbance during deployment and use^11^. To date, few eco-mooring trials have been conducted, with limited peer reviewed literature available on the subject, highlighting the novelty of the designs. Furthermore, few are undertaken in areas with large tidal ranges which pose additional threats to trials, and further stressors to the ecosystems; these include seabed exposure during low tide increasing the likelihood of seagrass entanglement and UV degradation of the meadows^10^.

Trials of eco-mooring systems undertaken in the UK are typically of Seaflex moorings, due to Seaflex already being an established UK provider and because of the design’s reported ability to endure variable tidal conditions. The trials have provided mixed results; with a Seaflex mooring installed in the waters surrounding Lundy island, showing positive results (although their effectiveness was deemed dependent on wave exposure and water depth)^10^, and in Mylor Harbour, Falmouth, UK, showing no improvement in the reduction of mooring scars, which was concluded a result of tidal influence^10^. Collectively, these studies emphasize the need for condition specific eco-moorings specifically designed for use in areas with a high tidal range.

To date most eco-mooring trials have been undertaken in Australia, in *Posidonia australis* meadows. These trials have had an overall higher success rate than those in the UK, which may reflect the reduced tidal ranges in the trial locations. A range of eco-mooring designs were tested and showed positive results against their traditional swing mooring counterparts. Screw moorings in Jervis Bay^12^, Ezyrider and Seaflex mooring systems all showed a considerable reduction in seagrass meadow scaring. The only design that showed negative results was a Cyclone seagrass friendly mooring, installed in Jervis Bay; which did little to reduce mooring scars^12^.

One downside associated with eco-moorings is the potential difficulty of finding an insurance policy to cover the system; a recent report by Amec Foster Wheeler Environment & Infrastructure UK Limited^13^ investigated the feasibility of using eco-moorings as management options for Marine Protected Areas (MPAs) in the UK. The study highlighted the lack of an established insurance market for the moorings. It was suggested that eco-moorings would fall under the definition of a swing mooring, and become insured under an existing policy, however the moorings may be assigned a premium for ‘new technology’ that insurers could be reluctant to cover or charge higher rates for.

In contrast to previous studies detailed above that assessed whether swing moorings could be replaced with new mooring designs, this study examines the effectiveness of simply modifying existing moorings. The study was designed to compare the impacts of a standard swing mooring and a modified swing mooring (“Stirling Mooring”, Community Seagrass Initiative) on seagrass density and blade length, species richness, species density, assemblage composition and sediment composition. The study was conducted in a dense seagrass meadow situated in the Salcombe ria, with seagrass typically growing to approximately 1.5 m in length in deeper parts of the channel, with shorter blades in shallower areas of the ria. We hypothesized that increased seagrass shoot density and blade length will be apparent proximal to the modified mooring, compared with the standard mooring, with recovery in areas absent of mooring chain disturbance over time. Significant differences in species assemblage between the moorings was predicted, with increased species richness and density apparent surrounding the modified mooring. Sediment composition was expected to reflect disturbance surrounding the standard mooring, with coarser particles sizes present. The development of this study, which demonstrates a viable mooring modification and quantifies the associated seagrass ecosystem recovery is fundamental in the evolution of seagrass conservation and management.

## Results

### Cost comparison for installation and maintenance

The mooring was modified at a total cost of £740 (£120 for modifications, £620 for new mooring tackle), which is considered to be a substantially lower cost than alternative eco-mooring designs on the market (cost model estimates of £1,620–£3,200 for components, and installation costs of £600^13^). There would be no anticipated additional maintenance costs for the modified mooring design than for a standard swing mooring, with annual checks required to monitor chain thickness and corrosion, with only additional buoy attachments to check and maintain. The modified mooring design also met the criteria of the existing insurance policy held by the Salcombe Harbor Authority^13^.

### Seagrass Shoot Density

**Figure 1:**
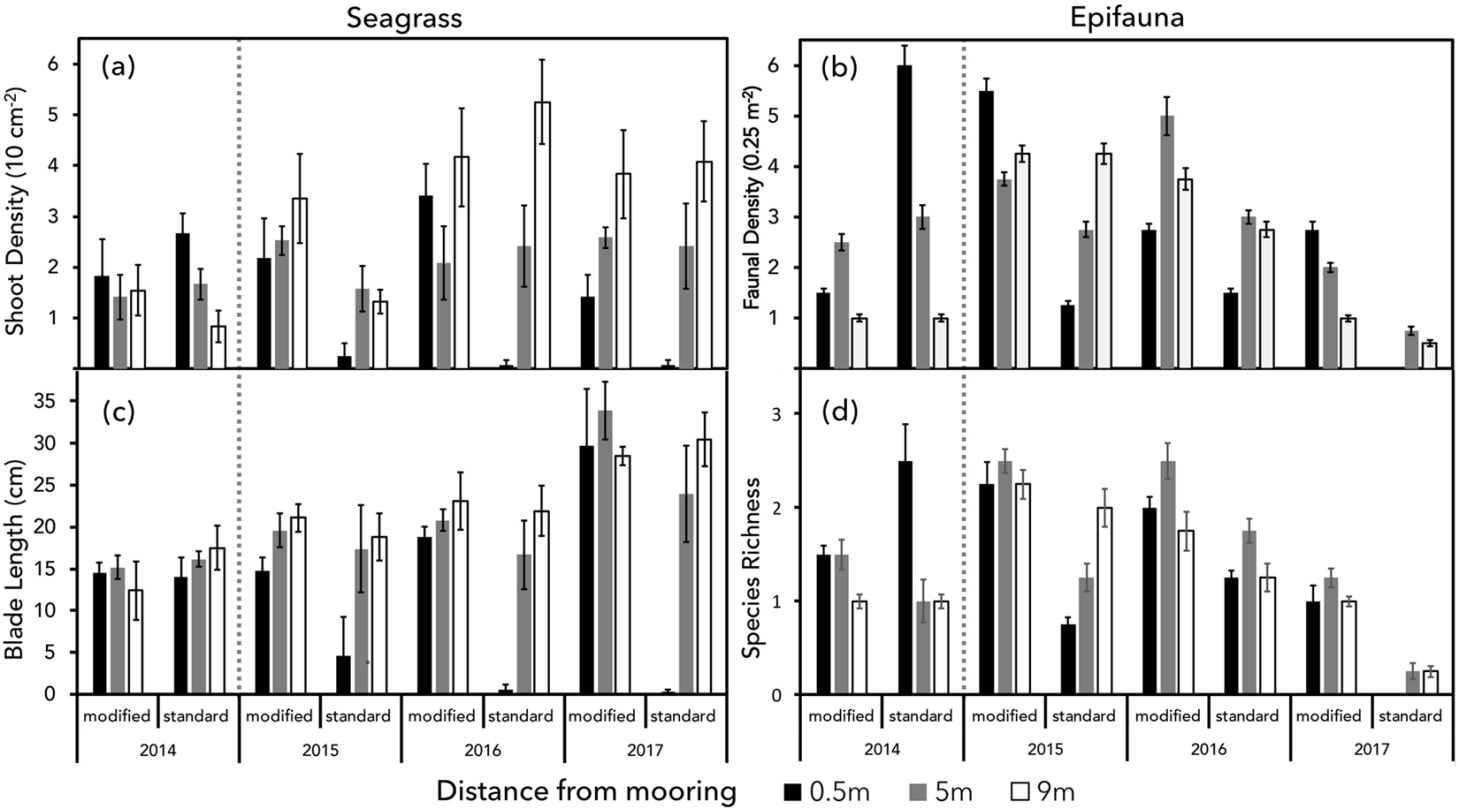
Seagrass (**a**,**c**) and epifauna (**b**,**d**) indicators before (2014) and after (2015–2017) installation of standard and modified moorings in a seagrass meadow, measured at increasing distance from the sinker block: (**a**) seagrass shoot density; (**b**) epifaunal abundance; (**c**) seagrass blade length; (**d**) epifaunal species richness

The average number of shoots in a 10 × 10 cm quadrat (0.01 m^2^) surrounding the standard mooring increased with distance from the sinker block across all years following installation (2015–2017). In the baseline year of 2014, the average number of shoots 0.5 m from the standard mooring sinker block was 2.16 ± 0.39 in 0.01 m^2^ (216 ± 39 m^−2^), with 1.66 ± 0.3 shoots in 0.01 m^2^ (166 ± 30 m^−2^) 5 m from the sinker block. Following the deployment of the standard mooring, the number of shoots at 0.5 m declined to 0.083 ± 0.08 in 0.01 m^2^ (8.3 ± 8 m^−2^) in 2017 and showed a slight increase 5 m from the block to 2.41 ± 0.8 shoots in 0.01 m^2^ (241 ± 84 shoots m^−2^) (Fig. 1a).

In comparison, the density of shoots surrounding the modified mooring showed some fluctuation, however, as expected no association with distance can be made. In the baseline year, the average number of shoots 0.5 m from the sinker block was 1.83 ± 0.7 shoots in 0.01 m^2^ (183 ± 72 m^−2^), with 1.41 ± 0.4 shoots in 0.01 m^2^ (141 ± 43 m^−2^) 5 m from the block. After the mooring installment, little change can be observed with 1.42 ± 0.4 (142 ± 44 m^−2^) shoots 0.5 m from the mooring, and 2.58 ± 0.2 shoots in 0.01 m^2^ (258 ± 20 m^−2^) 5 m from the mooring in 2017.

At 9 m from the sinker blocks of both moorings, treatments showed a slight incline in seagrass density over time; the standard mooring treatment increased from 0.83 ± 0.3 shoots in 0.01 m^2^ (83 ± 32 m^−2^) in 2014 to an average of 4.08 ± 0.79 shoots in 0.01 m^2^ (408 ± 79 m^−2^) in 2017, with the modified mooring treatment rising from 1.42 ± 0.5 shoots in 0.01 m^2^ (142 ± 50 m^−2^) to 3.83 ± 0.9 shoots in 0.01 m^2^ (383 ± 90 m^−2^).

Differences in seagrass density between treatments (p = 0.0068), distances (p = 0.0001) and years (p = 0.0001) were all statistically significant (Table 1). Pairwise tests conducted on the significant factors revealed a significant difference between the moorings 0.5 m from the sinker block (p = 0.0004), with an average shoot density of 0.64 ± 0.3 in 0.01 m^2^ (64 ± 30 m^−2^) 0.5 m from the standard mooring, and 2.21 ± 0.4 shoots in 0.01 m^2^ (221 ± 40 m^−2^) 0.5 m from the modified mooring.

**Table 1:** PERMANOVA examining differences in biological and physical parameters with year, mooring treatment, and distance from mooring, with pairwise tests for mooring treatments and distances where the main test showed a significant interaction. Simper analysis of species contribution to dissimilarity is also included. Bold type denotes a significant result.

Source	d.f	MS	F	P	Pairwise Comparison	F	P		
**Seagrass Density**									
Year (Yr)	3	8.481	5.8947	**0.0001**	Modified Mooring, 0.5 m	4.4474	**0.0004**		
Treatment (Tr)	1	10.845	7.5379	**0.0068**	Modified Mooring, 5 m	0.33883	0.7418		
Distance (Di)	2	20.81	14.464	**0.0001**	Modified Mooring, 9 m	0.62402	0.5304		
Yr x Tr	3	2.8841	2.0046	0.1208					
Yr x Di	6	8.0529	5.5971	0.0001					
Tr x Di	2	4.8466	3.3686	0.0392					
Yr x Tr x Di	6	2.9637	2.0599	0.0685					
Residual	72	1.4388							
Total	95								
**Seagrass Blade Length**									
Year	3	446.53	11.273	**0.0002**	Modified Mooring, 0.5 m	6.6133	**0.0001**		
Treatment	6	817.37	20.635	**0.0001**	Modified Mooring, 5 m	1.55	0.1342		
Distance	2	864.58	21.827	**0.0001**	Modified Mooring, 9 m	0.47481	0.6359		
Yr x Tr	6	216.31	5.461	**0.0022**					
Yr x Di	72	91.096	2.2998	**0.0418**					
Tr x Di	95	506.9	12.797	**0.0001**					
Yr x Tr x Di		75.005	1.8935	0.0943					
Residual	72	29.611							
Total	95								
**Sediment Particle Size**									
Treatment	1	14.143	12.196	**0.0042**	Modified Mooring, 0.5 m	6.4589	**0.0286**		
Distance	2	6.5242	5.626	**0.0156**	Modified Mooring, 5 m	1.3583	0.2522		
Tr x Di	2	4.2924	3.7015	**0.0444**	Modified Mooring, 9 m	0.35731	0.7974		
Residual	18	1.1597							
Total	23								
**Epifauna Diversity**									
Year	3	7.2877	9.1573	**0.0001**					
Treatment	1	10.714	13.463	**0.0005**					
Distance	2	0.36905	0.46372	0.6363					
Yr x Tr	3	1.0258	1.289	0.2862					
Yr x Di	6	0.83532	1.0496	0.3983					
Tr x Di	2	0.46429	0.5834	0.5638					
Yr x Tr x Di	6	0.35913	0.45126	0.8473					
Residual	60	0.79583							
Total	83								
**Abundance**									
Year	3	7.2877	9.1573	**0.0001**					
Treatment	1	10.714	13.463	**0.0005**					
Distance	2	0.36905	0.46372	0.6363					
Yr x Tr	3	1.0258	1.289	0.2862					
Yr x Di	6	0.83532	1.0496	0.3983					
Tr x Di	2	0.46429	0.5834	0.5638					
Yr x Tr x Di	6	0.35913	0.4126	0.8473					
Residual	60	0.79583							
Total	83								
**Assemblage**					**SIMPER Test**	**Av.Diss**	**Diss/SD**	**Contrib%**	**Cum %**
Year	3	5691	5.5277	**0.0001**	*Pagurus bernhardus*	39.26	1.27	51.32	51.32
Treatment	1	8118.4	7.8854	**0.0007**	*Gibbula umbilicalis*	22.76	0.91	29.75	81.07
Distance	2	1008.1	0.97919	0.4377	*Tritia reticulata*	7.86	0.5	10.27	91.34
Yr x Tr	3	1935.2	1.8796	0.0608	*Echinus esculentus*	3.36	0.35	4.4	95.74
Yr x Di	6	1151	1.1179	0.3323	*Macropodia spp*	1.4	0.22	1.83	97.58
Tr x Di	2	1293.2	1.2561	0.2862	*Pomatoschistus minutus*	0.61	0.22	0.8	98.37
Yr x Tr x Di	6	695.67	0.6757	0.8198	*Maja brachydactyla*	0.6	0.21	0.78	99.15
Residual	60	1029.5			*Calliostoma zizyphinum*	0.32	0.15	0.42	99.58
Total	83								

### Seagrass Blade Length

At the standard mooring treatment, there was a general increase in blade length with distance from the sinker block; in the baseline year of 2014 (prior to mooring deployment), the mean blade length measured 14.02 ± 2.3 cm at a distance of 0.5 m from the sinker block, and 16.15 ± 0.9 cm at 5 m from the block. Three years after the deployment of the standard mooring, the mean blade length had dropped to 0.25 ± 0.3 cm at 0.5 m from the block and increased to 23.95 ± 5.8 cm at 5 m from the sinker block (Fig. 1c).

The modified mooring treatment blade length remained relatively stable across all distances, whilst showing an increase in blade length over time (2014–2017). In the baseline year of 2014, the mean blade length measured 14.5 ± 1.2 cm at 0.5 m, and 15.18 ± 1.4 cm 5 m from the sinker block. 3 years after deployment, the average blade length of the modified mooring measured 29.72 ± 6.8 cm 0.5 m from the sinker block and 33.9 ± 3.4 cm 5 m from the block (Fig. 1c). Quadrat samples 9 m from the sinker block (away from influence from the chain) remained relatively stable over time, with an increase observed in 2017 in both conditions. Within the standard mooring treatment, 9 m from the sinker block, a mean blade length of 17.51 ± 2.9 cm was observed in 2014, which increased to 30.5 ± 3.2 cm in 2017, and a blade length of 12.41 ± 3.5 cm in 2014 was observed in the modified mooring treatment, which increased to 28.47 ± 1.1 cm in 2017.

Observed differences in blade length between the treatments (p = 0.0001), distances (p = 0.0001) and over time (p = 0.0002) were significant (Table 1). Pairwise tests between the significant factors revealed a significant difference between the moorings 0.5 m from the sinker block, with an average blade length of 4.86 ± 1.9 cm 0.5 m from the standard mooring and 19.46 ± 2.3 cm 0.5 m from the modified mooring.

### Sediment Particle Size

**Figure 2:**
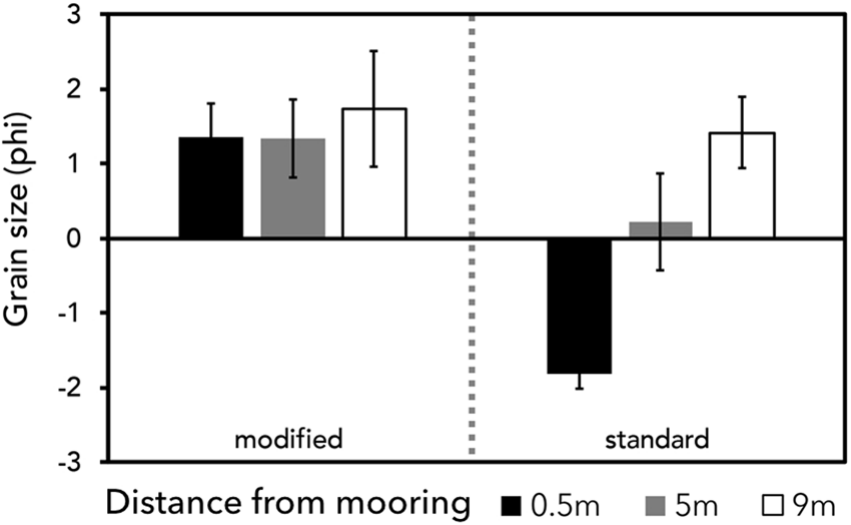
Sediment particle sizes (phi) at increasing distances from each mooring treatment (Udden-Wenworth scale).

Grain size distribution at the standard mooring was very poorly sorted, dominated by medium to fine sand (53.3%) and fine to coarse gravel (40.8%) (Fig. 2). Mean grain sizes were shown to decrease with distance from the sinker block; samples taken at 0.5 m had a mean phi grain size of −1.807 φ, (very fine to fine gravel on the Udden-Wentworth scale), which decreased to 0.221 φ (coarse sand) 5 m from the block.

The modified mooring treatment was poorly sorted, with the sample dominated by fine to medium sand (72.8%) (Table 1; Fig. 2). The samples showed minimal fluctuations in grain size with distance, with grain sizes of 1.357 φ at 0.5 m and 1.339 φ 5 m from the block (medium sand, medium sand).

Quadrats located away from chain abrasion (9 m) showed similar grain sizes; with an average grain size of 1.415 φ (medium sand) 9 m from the standard mooring, and 1.739 φ (medium sand) 9 m from the modified mooring.

These differences in grain size distribution between treatments were statistically significant (p = 0.0042, p = 0.0156) (Table 1). Pairwise testing between the significantly different factors showed a significant relationship between the modified and standard mooring treatments 0.5 m from the sinker block (p = 0.0286), with the standard mooring having a mean grain size of −1.807 ± 1.8 φ (very fine to fine gravel), and the modified mooring with 1.357 ± 0.5 φ (medium sand) 0.5 m from the sinker block.

### Faunal Density

Epifaunal density surrounding the standard mooring increased with distance from the mooring sinker block and showed an overall decline over time (2015–2017).

Prior to the deployment of the moorings (2014), the standard mooring had an average abundance of 6 ± 0.4 individuals 0.5 m from the sinker block, and 3 ± 0.2 5 m from the block. Following the deployment of the standard mooring, the average number of individuals per 0.25 m2 quadrat declined to 0, 0.5 m from the mooring sinker block in 2017, and 0.75 ± 0.08 5 m from the block (Fig. 1b).

The average number of individuals per quadrat surrounding the modified mooring also showed variation over time, although little relationship with distance can be observed. In the baseline year of 2014 the modified mooring had an average abundance of 1.5 ± 0.09 individuals 0.5 m from the sinker block, and 2.5 ± 0.2 5 m from the block. After the deployment of the modified mooring the species abundance increased in the years 2015 and 2016, peaking in 2015 0.5 m from the block at 5.5 ± 0.2 individuals, followed by a decline in 2017 to 2.75 ± 0.2 individuals 0.5 m from the sinker block, and 2 ± 0.09 individuals 5 m from the block (Fig. 1b). Despite this, the average faunal density remained consistently higher surrounding the modified mooring than the standard mooring post deployment.

Quadrat samples 9 m from the sinker block (away from influence of the chain) showed low faunal density in 2014 for both treatments (standard, modified, 1 ± 0.08, 1 ± 0.08), followed by an increase in faunal density with both samples peaking in 2015 (standard, modified, 4.25 ± 0.2, 4.25 ± 02), and declining in 2017 (standard, modified, 0.5 ± 0.1, 1 ± 0.1) (Fig. 1b).

### Species Richness

The number of species surrounding the standard mooring treatment was shown to fluctuate over time following the deployment of the moorings (2015–2017), with the average number of species 0.5 m from the sinker block remaining consistently lower than quadrats 5 m and 9 m from the sinker block.

Prior to the deployment of the moorings (2014), the standard mooring had an average species richness of 2.5 ± 0.4 species per quadrat 0.5 m from the sinker block, and 1 ± 0.2 species 5 m from the block. Following the deployment of the moorings, the average species richness surrounding the standard mooring dropped to 0 species 0.5 m from the mooring sinker block in 2017, and 0.25 ± 0.08 species 5 m from the block (Fig. 1d).

The average species richness surrounding the modified mooring also fluctuated over time, whilst remaining consistently higher than the standard mooring across all distances.

In the baseline year of 2014, the modified mooring had an average species richness of 1.5 ± 0.09 per quadrat, 0.5 m from the block, and 1.5 ± 0.2, 5 m from the mooring sinker block. Three years after the mooring deployment (2017), the average number of species at 0.5 m from the sinker block had declined to 1 ± 0.2 species per quadrat, and 1.25 ± 0.1 species at 5 m from the block (Fig. 1d).

The average species richness 9 m from the standard mooring sinker block (away from chain disturbance) showed a slight decline over time, from 1 ± 0.08 species per quadrat in 2014, to 0.25 ± 0.05 species in 2017.

The species richness 9 m from the modified mooring peaked at 2.25 ± 0.2 species in 2015, then dropped to 1 ± 0.06 species in 2017.

### Assemblage

**Figure 3:**
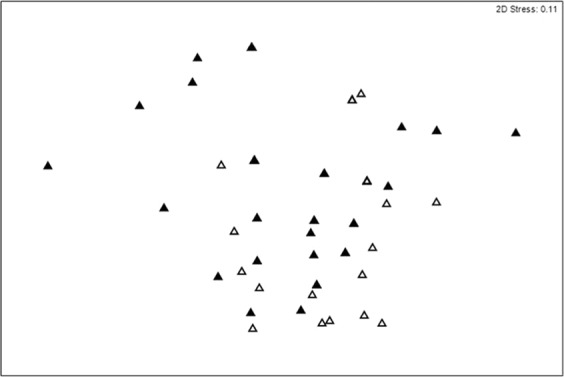
Multidimensional scaling plot based on Bray-Curtis similarity resemblance matrix of epifaunal assemblages around the standard (filled) and modified (unfilled) mooring treatments.

The assemblage composition significantly differed between mooring Treatment (p = 0.0007) and Year (p = 0.0001) (Table 1). The assemblage composition was more dispersed for the standard mooring than the modified mooring (MVDISP: Standard 1.091, Modified 0.909, Fig. 3). The species driving the differences between treatments were *Anemonia viridis*, *Pagurus* spp. and *Gibbula umbilicalis*. *G. umbilicalis* and *Calliostoma zizyphimum* were the only species with greater abundance in the standard mooring compared to the modified mooring, two species were found in similar abundances between treatments (*Tritia reticulata*, *Pomatoschistus minutus*), while the majority (six species: *Anemonia viridis*, *Pagurus bernhardus*, *Echinus esculentus*, *Macropodia* spp, *Maja brachydactyla*) were found in greater abundances in the modified mooring.

## Discussion

This study confirms the negative impacts that standard swing moorings can have on sensitive seagrass ecosystems^2^, but also shows that these impacts can be mitigated through simple modification of existing moorings. A new and relatively simple modification to a swing mooring has proven successful in the reduction of chain contact with the seafloor, leading to reduced environmental impacts across multiple biological and physical parameters. The study was conducted within an established dense seagrass meadow in the Salcombe ria; the data collected indicates the Salcombe ria seagrass meadow to be a typical dense meadow, with species density, richness and assemblage characterizing the typical ecology representative of a seagrass meadow in the UK.

The mooring modifications and installation costs were considered substantially lower than for alternative designs, costing a minimum of 67% less than alternative eco-mooring designs on the market (cost model estimates of £1,620 - £3,200 for components, and installation costs of £600^13^). The modified mooring design also met the criteria for the existing insurance policies^13^ suggesting that a modified swing mooring design may instill increased confidence in insurance companies, due to their confidence in the traditional swing mooring. It is suggested, that alongside a targeted educational program directed towards regulators and the public, reduced costs and the availability of insurance policies for the moorings, the public would be encouraged to modify traditional swing moorings to reduce mooring impacts on seagrass beds.

The study found that seagrass density and blade length both increase with distance from the standard mooring as hypothesized, with a weaker correlation observed in the modified mooring treatment. This indicated substantially reduced scouring impacts on seagrass in the modified mooring treatment, compared to the standard mooring. An increase with distance in both parameters was still evident in the modified mooring treatment, however to a significantly lessened extent. Seagrass recovery was evident over time (2014–2017) in the modified mooring treatment (after modification of a swing mooring) in blade length and faunal density, indicating that the replacement of current swing moorings could reduce fragmentation of seagrass meadows caused by moorings, and encourage recoverability of the ecosystem.

The highest degree of impact across both parameters (density, blade length) was observed 0.5 m from the standard mooring sinker block, as hypothesized. A lessened degree of disturbance 5 m from the block was also observed, with minimal disturbance at 9 m indicating that any acute impacts on the seagrass meadows from the standard mooring were localized. These results are reflected in a study by Unsworth, *et al*.^2^, who observed a similar linear gradient with 79% of quadrats located 0 m from a swing mooring containing no seagrass. Unsworth, *et al*.^2^ documented impacts up to 20 m from the mooring in the study, suggesting a larger impact area beyond the extent of the mooring chain and scarring area. However, despite this, seagrass degradation as a result of mooring impacts appears to occur on a localized scale, this is considered substantial due to damage occurring in the center of the seagrass meadows, often resulting in habitat fragmentation reducing the resilience of seagrass to additional stressors.

Sediment grain size distributions supported the hypotheses suggesting significantly different sediment compositions between treatments (S.D p < 0.05); the sample closest to the standard mooring showed coarser grain sizes, with finer grains in locality to the modified mooring as predicted. Disturbance from mooring chains scouring the seafloor has the potential to resuspend small grains, modifying the sediment composition favoring larger grain sizes such as shell fragments and gravel^6^. The resuspension of fine particles can also increase water turbidity, reducing sunlight and consequently seagrass photosynthesis and growth^14^.

Changes in sediment composition can also be linked to seagrass density, with reduced density resulting in a lack of sediment trapping and retention, leading to coarser sediment compositions^15^. In the present study, the sediment particle sizes 0.5 m from the standard mooring in 2017, in areas of low seagrass density were coarser than in quadrats 9 m from the mooring sinker block in areas of increased density, suggesting seagrass density may have had an influence on sedimentation rates. A similar relationship was also observed between sediment size and seagrass blade lengths 0.5 m from the moorings, with finer sediment particle sizes and longer blade lengths local to the modified mooring, compared to the standard mooring, suggesting that long seagrass blades may trap fine sediment particles, leading to an increase in sedimentation in the area. Increased sediment deposition in seagrass meadows, encourages the sequestration of organic carbon, contributing to the reduction of greenhouse gases^15,16^.

It is important to note that factors such as coastal development and land use changes can also influence sediment sizes and composition, and the extent of influence would need further research. However, the current study appears to show strong correlations between mooring disturbance, sediment changes and seagrass density and blade length.

Overall, the findings indicate a high degree of disturbance surrounding the standard mooring, compared with the modified mooring, which showed little impact on the surrounding sediment. Species richness, density and assemblage were found to be statistically different between treatments as hypothesized, suggesting a difference in habitat or ecological features of the sites. Increased species richness and abundance surrounding the modified mooring were evident, implying greater biodiversity supported by increased density and blade length of seagrass surrounding the modified mooring.

Similar findings were found by McClosky and Unsworth^6^, who reported increased faunal density and species richness in areas of high seagrass cover. Bowden *et al*.^17^ and Collins *et al*.^18^ also found a decline in species richness and density in unvegetated mooring scars.

A decline in species richness and density surrounding the standard mooring may be a result of species preference for high density seagrass, which offers increased cover from predators; McClosky and Unsworth^6^ suggested that species such as Plaice were found to prefer bare substrate, due to difficulty locating prey in dense seagrass meadows.

Moreover, the effects of interspecies and intraspecies competition must be acknowledged as an influential factor in changes in species richness, this could be emphasized as a result of increased species concentration in seagrass meadow fragments^19^.

Species habitat preferences may also have influenced the observed differences in species compositions between the mooring treatments. McClosky and Unsworth^6^ suggested independent species preferences for bare or vegetated substratum, with observations of Sand Gobies and Plaice preferring to inhabit areas of bare substratum, whereas many juvenile commercial fish species showed a preference for dense seagrass meadows.

It is worth noting that this study was conducted in a single seagrass bed, with only one experimental unit of each mooring type, therefore it is recommended that further spatially replicated experiments are undertaken, in order to confirm the results of the current study. The challenge now is to convince managers and boat owners to modify their swing chain moorings to enable damaged seagrass meadows to recover and restore their associated ecosystem services. Local targeted education programs for regulators and the public could help to raise awareness about the importance of seagrass meadows, the damage that is being caused and how a simple modification to moorings can result in positive recovery for this important habitat. In addition, statutory legislation should be implemented to reduce further human induced degradation of seagrass meadows worldwide.

## Conclusion

The current study demonstrates a cost-effective approach to reduce mooring impacts in seagrass meadows and highlights the destructive potential of traditional swing mooring systems.

In contrast to previous studies describing new ‘eco-mooring’ designs, this paper has offered a low-cost approach through the modification of an already existing swing mooring. The modified mooring successfully reduced chain abrasion of the seafloor, using floats to lift the mooring chain off the seabed at low tide, and greatly reduced the associated negative impacts on the seagrass ecosystem without compromising the integrity of the mooring.

## Methods

The study site was situated in the Salcombe ria, UK, chosen because of its combination of established dense seagrass meadows skirting the channel, and intense boating activity all year round. The site has a strong tidal influence, with a tidal range of 5.5 m and a depth of 10 m in the deeper parts of the channel. The experimental treatments, a modified swing mooring and a standard swing mooring, were located 76 m from the shore, and 60 m apart, and installed at low tide on the 18th April 2014. The alterations to the mooring cost £120, in addition to this, mooring tackle was replaced at a maximum cost of £620. Maintenance requirements for the mooring include monitoring chain thickness, corrosion and buoy attachments, with associated costs predicted to align with those for standard swing mooring designs.

### Treatment Descriptions

**Figure 4:**
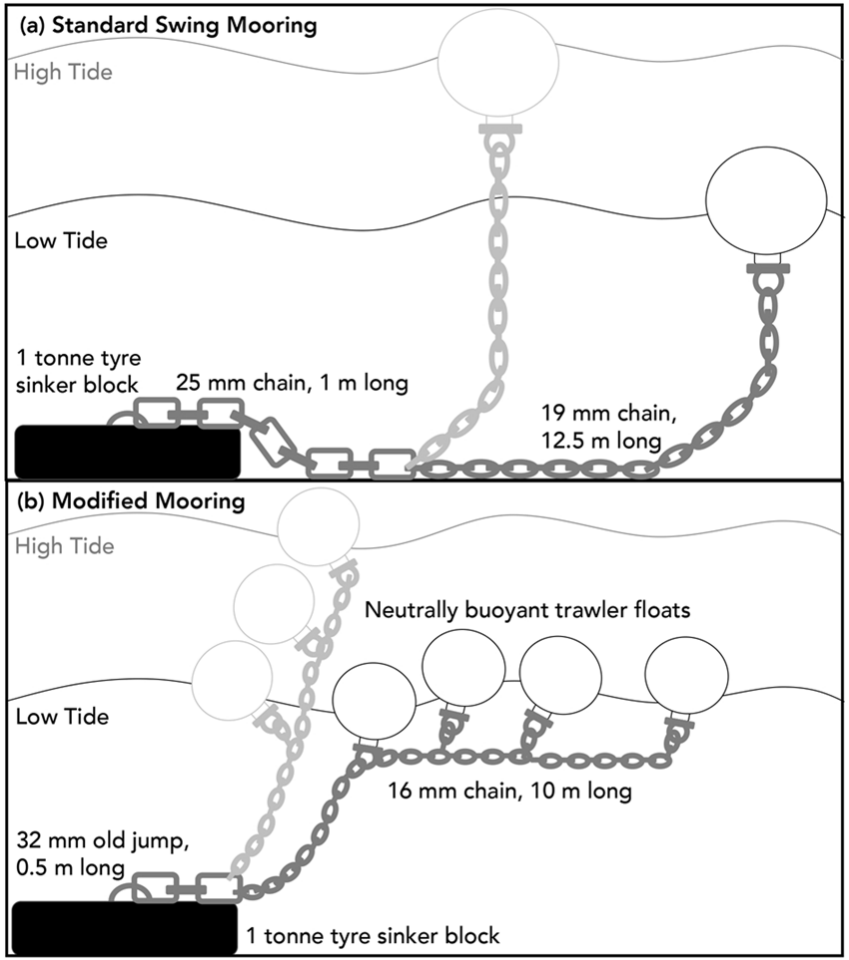
Diagram of a standard mooring **(a**) and modified mooring (**b**) treatments, showing position of the mooring floats and chains at high tide (light grey) and low tide (dark grey).

The first treatment comprised of a standard swing mooring, reinstalled in 2014. The mooring consisted of a 1 tonne tyre sinker block and eye, placed on the seafloor, with 1 m of 25 mm stainless-steel chain leading off it. The chain was shackled to a light 19 mm chain, which reached 12.5 m from the sinker block, and was shackled to a 90 cm surface mooring buoy (Fig. 4).

The second treatment was a swing mooring, modified to reduce impact on the seafloor from the stainless-steel chain. The mooring was configured of a 1 tonne tyre block and eye, with 0.5 m of 32 mm Old Jump that rests on top of the block. Leading off this was 10 m of 16 mm chain, shackled to which were trawler floats, which kept the chain elevated during high and low tides (Fig. 4).

### Sampling Procedure

Data for this study were collected through the citizen science project, the Community Seagrass Initiative^20^. To limit potential inconsistencies between divers, all Community Seagrass Initiative volunteers were subject to training beforehand.

Measurements of seagrass shoot density, blade length, and faunal density were collected around each mooring by a team of 5 dive pairs. Measurements of each variable were taken at three distances along a transect from the sinker block: 0.5 m, 5 m and 9 m. Each transect was replicated across four bearings of NE, SE, SW and NW, providing four replicates at three distances from each mooring. Data were collected from 2014 to 2017 in March of each year (to eliminate seasonal influence) except for 2017, when collections were delayed until May because of poor diving conditions. The distance of 0.5 m represented a zone of direct impact, 5 m the near impact zone, and 9 m, an area away from any influence from the chain.

At each sampling location, a 0.25 m^2^ quadrat was placed over the transect line, and photographed after any disturbed sediment had settled, using a Gopro Hero 4 camera.

The following parameters were recorded in the field:

1. The length of 10 haphazardly selected seagrass blades (cm) within a 0.25 m^2^ quadrat.
2. The number of seagrass shoots in 3 random 0.01 m^2^ squares of the quadrat.

In 2017, additional parameters were investigated through the collection of sediment samples; samples were collected in 125 ml sample pots from the center of the quadrat, which were then sealed and chilled in a lab at 4 °C for further analysis.

### Sediment Sample Particle Size Analysis

Sediment particle size analysis was undertaken in accordance to the NMBAQC’s Best Practice Guidance for Marine Sediments^21^. Samples were mixed thoroughly, and subsamples of approximately 5 ml of the sample obtained with a spatula. Material >1 mm and <1 mm was separated using a 25 mm diameter 1 mm sieve, a vial funnel and a 12 ml vial. A pressurized water spray was used to aid this process.

For each sample, 5 replicate vials were made, and placed methodically in a sampling rack, with the vial locations noted. The sampling rack was then placed in the Malvern Mastersizer 2000 (general analysis model with irregular particle shape and enhanced sensitivity, reference index of 1.53) for laser diffraction. The instrument was set to run 5 replications on each sample.

Samples were refrigerated until settlement had occurred, and any excess surface water was drained from them. For each sample, a 250 ml and a 100 ml beaker were assigned labels by proxy. The 250 ml beakers were weighed to 2 dp and noted. Approximately 30 ml of the sample was sieved through a 1 mm mesh into a funnel held over the 250 ml beaker. A small sieve brush and a fine water spay were used to aid the sieving process, depositing material <1 mm into the 250 ml beaker. Any sediment >1 mm left on the surface on the sieve was deposited into the 100 ml beaker. Both beakers (250 ml and 100 ml) were then dried in an oven for 48 hours at 105 °c.

Following this, the 250 ml beaker was then reweighed, to determine the weight of the material <1 mm. The material in the 100 ml beakers was dry sieved using 16 mm to 1 mm sieves, at half phi intervals, and the weights recorded.

### Epifaunal Analysis

Images taken of the quadrats were analyzed alongside diver observations, and epifaunal species identified to the lowest taxonomic level. Both sessile and mobile epifauna were recorded, and the species richness and density per quadrat noted.

### Statistical Analysis

Statistical analysis was conducted using PRIMER 7 with PERMANOVA^22,23^. The threshold for determining statistical significance was set at P < 0.05. Variability of the data is reported as standard error about the mean.

The data for variables seagrass blade length, shoot density, and sediment composition were first calculated and arranged into a resemblance Euclidian distance matrix to show the similarity or dissimilarity between each pair of data, as coefficients. Permutational multivariate analysis of variance (PERMANOVA) was used to determine differences in variables. Pairwise tests were then conducted on the statistically significant variables to identify where the differences occurred.

The epifaunal data were subject to resemblance testing, for faunal density, species richness and assemblage variables, using the Bray Curtis technique. A dummy variable of 1 was assigned to the data to aid distinction between the treatment groups. Multivariate dispersion (MVDISP) was then used to assess dispersion of the significant factors, and the resemblance matrix data visualized in an nMDS (non-metric Multi-Dimensional Scaling) plot, providing a graphical representation of how the variables relate to one another. Next PERMANOVA tests were then performed on the resemblance data, to determine the statistical significance of the data. The statistically different (P ≤ 0.05) factors were then further analyzed with SIMPER tests, to identify the discriminating species between the treatment (modified mooring and standard mooring) and year (2014, 2015, 2016, 2017) factors.

## Data Availability

The datasets generated during and analyzed during the current study will be archived in the Marine Biological Association repository (DASSH, The Archive for Marine Species and Habitats Data), and made available via the MEDIN (Marine Environmental Data and Information Network) portal (https://portal.medin.org.uk/portal/start.php).
